# Environmental exposures and child and maternal gut microbiota in rural Malawi

**DOI:** 10.1111/ppe.12623

**Published:** 2020-02-03

**Authors:** Emma Kortekangas, Arox W. Kamng'ona, Yue‐Mei Fan, Yin Bun Cheung, Ulla Ashorn, Andrew Matchado, Basho Poelman, Kenneth Maleta, Kathryn G. Dewey, Per Ashorn

**Affiliations:** ^1^ Center for Child Health Research Faculty of Medicine and Health Technology Tampere University Tampere Finland; ^2^ Department of Biomedical Sciences College of Medicine University of Malawi Blantyre Malawi; ^3^ Program in International and Community Nutrition University of California Davis Davis CA USA; ^4^ Program in Health Services & Systems Research and Centre for Quantitative Medicine Duke‐NUS Medical School Singapore Singapore; ^5^ School of Public Health and Family Medicine University of Malawi College of Medicine Blantyre Malawi; ^6^ Department of Nutrition University of California Davis Davis CA USA; ^7^ Department of Paediatrics Tampere University Hospital Tampere Finland

**Keywords:** child health, environment, gut microbiota, seasons, socio‐economic factors

## Abstract

**Background:**

Gut microbiota composition is associated with child health, but the effect of the environment on microbiota composition is not well understood. Few studies have been conducted in low‐income settings where childhood malnutrition is common and possibly related to microbiota composition.

**Objectives:**

To investigate whether gut microbiota composition in young children and their mothers is associated with different environmental exposures in rural Malawi. We hypothesized that more adverse environmental exposures would be associated with lower levels of microbiota maturity and diversity.

**Methods:**

Faecal samples from up to 631 children and mothers participating in a nutrition intervention trial were collected at 1, 6, 12, 18, and 30 months (children) and at 1 month (mothers) after birth and analysed for microbiota composition with 16S rRNA sequencing. Bacterial OTU and genus abundances, measures of microbiota maturity and diversity, and UniFrac distances were compared between participants with different environmental exposures. The exposure variables included socio‐economic status, water source, sanitary facility, domestic animals, maternal characteristics, season, antibiotic use, and delivery mode.

**Results:**

Measures of microbiota maturity and diversity in children were inversely associated with maternal education at 6, 18, and 30 months and did not otherwise differ consistently between participants with different environmental exposures. Phylogenetic distance was related to season of stool sample collection at all time points. At the level of individual OTUs and genera, season of stool sample collection, type of water source, and maternal education showed most associations with child gut microbiota, while HIV status was the most important predictor of relative OTU and genus abundances in mothers.

**Conclusion:**

The results do not support the hypothesis that adverse environmental exposures are broadly associated with lower microbiota maturity and diversity but suggest that environmental exposures influence the abundance of several bacterial OTUs and genera and that low maternal education is associated with higher microbiota maturity and diversity.

**Synopsis**

1 **Study question**

Are environmental exposures associated with the composition of children's and their mothers’ gut microbiota in rural Malawi?

2 **What's already known**

Gut microbiota composition is associated with child health, and it is influenced by several external factors including birth mode and infant feeding. The environment is also believed to contribute to inter‐individual differences in microbiota composition, and there are considerable differences in the microbiota composition of people living in different geographic areas.

3 **What this study adds**

In the studied Malawian population, microbiota maturity and diversity in children were associated with low maternal education but not with other exposure variables. Several environmental exposure variables, including season of stool sample collection, maternal education, water source, and domestic animals, showed associations with phylogenetic distance between samples and relative abundances of individual bacterial genera and taxa. The largest number of associations was in children observed for season, maternal education, and water source, whereas in mothers, most differences were related to HIV status.

## 1 BACKGROUND

The composition of the gut microbiota has been associated with various health outcomes, including allergic diseases and nutritional status.^1^, ^2^ Especially in early life while the microbiota is developing, changes in its composition can be influential and contribute to undernutrition in resource‐poor environments.^3^, ^4^ The human gut changes considerably during the first 2 years of life as children progress from breast milk‐dominated diets and are exposed to greater numbers of bacterial species.^5^, ^6^ Undernourished children have been reported to have gut microbiota that appear less mature than those of chronologically age‐matched healthy children.^7^ Preclinical evidence that this immaturity is causally related to undernutrition comes from studies of gnotobiotic mice that have been colonized with microbial communities from undernourished and healthy Malawian children and fed diets representative of those of the donors; recipients of immature microbiota exhibited reduced growth rates and associated metabolic abnormalities.^8^

While there is growing evidence on the association between microbiota composition and child health, the determinants of gut microbiota composition are only partly understood. Mode of delivery, infant feeding practices, diet, and age are among the most important factors known to affect the composition of the developing gut microbiota.^5^, ^9^, ^10^, ^11^ Furthermore, socio‐economic characteristics, maternal marital status, number of siblings, and exposure to furry animals may contribute to inter‐individual differences.^12^, ^13^, ^14^, ^15^ Water and sanitation are associated with child health outcomes, but their effect on the microbiota warrants further investigation.^16^ There are considerable differences in the microbiota composition of people living in different geographic areas, and within populations, seasonal variations have been observed.^5^, ^11^, ^17^, ^18^ So far, there is limited knowledge on the effects of environmental exposures on the gut microbiota in low‐resource settings; in conditions where childhood malnutrition is common and associated with microbiota immaturity.^19^

The aim of the present study was to investigate whether gut microbiota composition in young children and their mothers is associated with different environmental exposures. We analysed data from children and mothers who were followed up during pregnancy and 30 months after birth as part of the iLiNS‐DYAD trial in Malawi. iLiNS‐DYAD was a nutrition intervention trial conducted by the International Lipid‐Based Nutrient Supplements Project study team (iLiNS‐DYAD‐M; NCT01239693). The main objective of the study was to determine the effect of a lipid‐based nutrient supplement (LNS) on foetal and child growth.^20^, ^21^ Socio‐economic data and biological samples were collected at several time points. We hypothesized that more adverse environmental exposures would be correlated with lower levels of microbiota maturity and diversity in mothers and their children at the age of 18 and 30 months. In addition, we analysed differences in the relative abundances of specific bacterial OTUs and genera between children with different levels of environmental exposures and associations between the environment and maternal microbiota.

## 2 METHODS

### 2.1 Cohort selection

The iLiNS‐DYAD trial during which the data for this study were collected was a randomized, controlled, and partly blinded clinical trial that was conducted in the Mangochi district in southern Malawi. The study area is mostly rural with one semi‐urban area and has a high prevalence of child undernutrition and high fertility rates. The study enrolled 1391 pregnant women with ultrasound confirmed pregnancy of no more than 20 completed gestation weeks who were permanent residents of the study catchment area.

### 2.2 Faecal sample collection and processing

Faecal samples from children were collected at home visits at 1, 6, 12, 18, and 30 months of age and from mothers at 1 month after delivery (Figure S1). If a participant had diarrhoea, no sample was collected and the visit was postponed by 2 weeks. Samples that had been placed in collection containers by mothers were picked up by research assistants on the same day and placed in cooler bags, after which time they were transferred to cryovial tubes for storage and frozen at −20°C within approximately 6 hours. Within 48 hours, samples were transported to a central laboratory where they were frozen at −80°C and stored for up to 6 months until being shipped to the analysis laboratory on dry ice.

Microbiota data were obtained from the above‐mentioned faecal samples using previously described DNA extraction and high‐throughput sequencing methods.^7^, ^8^, ^22^ The relative abundance of each OTU in each sample was quantified by the number of 16S rRNA sequence reads assigned to it. To exclude artefacts, OTUs were filtered with a threshold of 0.1% of sequencing reads in at least two samples. The V4‐16S sequence data generated and analysed for this study are available through the European Nucleotide Archive under the study accession number PRJEB29433.

### 2.3 Outcomes

Different measures capturing specific aspects of microbiota composition were used as outcome variables. Variables measuring microbiota maturity and diversity in children at 18 and 30 months were used as primary outcomes. Additionally, we performed secondary analyses with microbiota outcomes at 1, 6, and 12 months in children and 1 month after delivery in mothers to assess whether the effect of environmental exposures on child microbiota composition increases with age as children become more mobile and start consuming complementary foods and whether these effects are also observed in their mothers. Other secondary outcomes included variables measuring phylogenetic distances between samples and the relative abundance of individual bacterial OTUs and genera.

### 2.4 Exposures

Predictor variables included measures of environmental exposures and other factors that could influence microbiota composition based on previous studies and biological plausibility. As a proxy for socio‐economic status, a previously described household assets index was constructed by principal component analysis based on ownership of a set of assets (radio, television, cell phone, bed, mattress, bed net, and bicycle), lighting source, drinking water source, sanitary facility, and flooring materials.^23^ Having a household assets index below the sample median was considered an adverse environmental exposure, as was the presence of any chickens, goats, or cows in the household, not having piped drinking water, having no sanitary facility or a regular pit latrine, maternal education below the sample median (in years of primary and secondary school), maternal HIV, and maternal marital status other than married. Because delivery mode, duration of exclusive breast feeding, season, age, and antibiotic use have been shown to be associated with microbiota composition, these variables as well as child sex, maternal age, household crowding (in number of people living in the household), sample processing pool, and 16S rRNA sequencing depth were analysed as secondary exposures.^5^, ^12^, ^18^, ^24^

### 2.5 Statistical analysis

Separate linear models for each time point were used to test the hypotheses that environmental exposures are associated with decreased microbiota maturity (MAZ) and diversity (Shannon's diversity index). Analyses were performed with multivariable models that included all environmental exposure variables and secondary exposures. Because the participant's intervention group in the iLiNS‐DYAD trial may modify the association between the environmental exposure variables and the outcomes of interest, an interaction term was added to the multivariable models to test for interaction between the trial intervention and each exposure variable. Additionally, we examined confounding by gestational age at delivery at all time points in the child samples.

Permutational multivariate analysis of variance (PERMANOVA) models using both weighted and unweighted UniFrac distances were used to test whether participants with similar environmental exposures had microbiota compositions more similar to each other than to those of participants with different exposures.^25^

Differences in bacterial relative abundances were analysed at OTU and genus level. Differences in CSS‐normalized counts of specific OTUs were tested with multivariable zero‐inflated negative binominal models. All predictor variables were included in the analysis. Differences in all OTUs that had non‐zero counts in at least 20% of all samples were analysed, and FDR‐corrected *P*‐values were calculated. Differences in the relative abundances of the 23 most common genera were tested by assessing associations between predictor variables and OTU counts aggregated to genus level using multivariable regression models with robust standard errors.

### 2.6 Missing data

If outcome data were missing for a participant at a certain time point, the participant was excluded from the respective analyses. For exposure variables, imputation was deemed necessary for a group of exposure variables if their combination resulted in more than 10% dropped observations. If a single exposure variable had more than 20% missing values, it was excluded from the analysis. In addition, we conducted a sensitivity analysis where all exposure variables regardless of number of missing values were imputed with 50 imputations using chained equation methods.

### 2.7 Ethics approval

The iLiNS‐DYAD study was approved by the College of Medicine Research and Ethics Committee (COMREC) in Malawi and the ethics committee of the Pirkanmaa hospital district in Finland. All participants provided informed consent at enrolment by signing or thumb printing a consent form.

For further details on methods, see Methods S1.

## 3 RESULTS

### 3.1 Sample formation and characteristics

**Figure 1:**
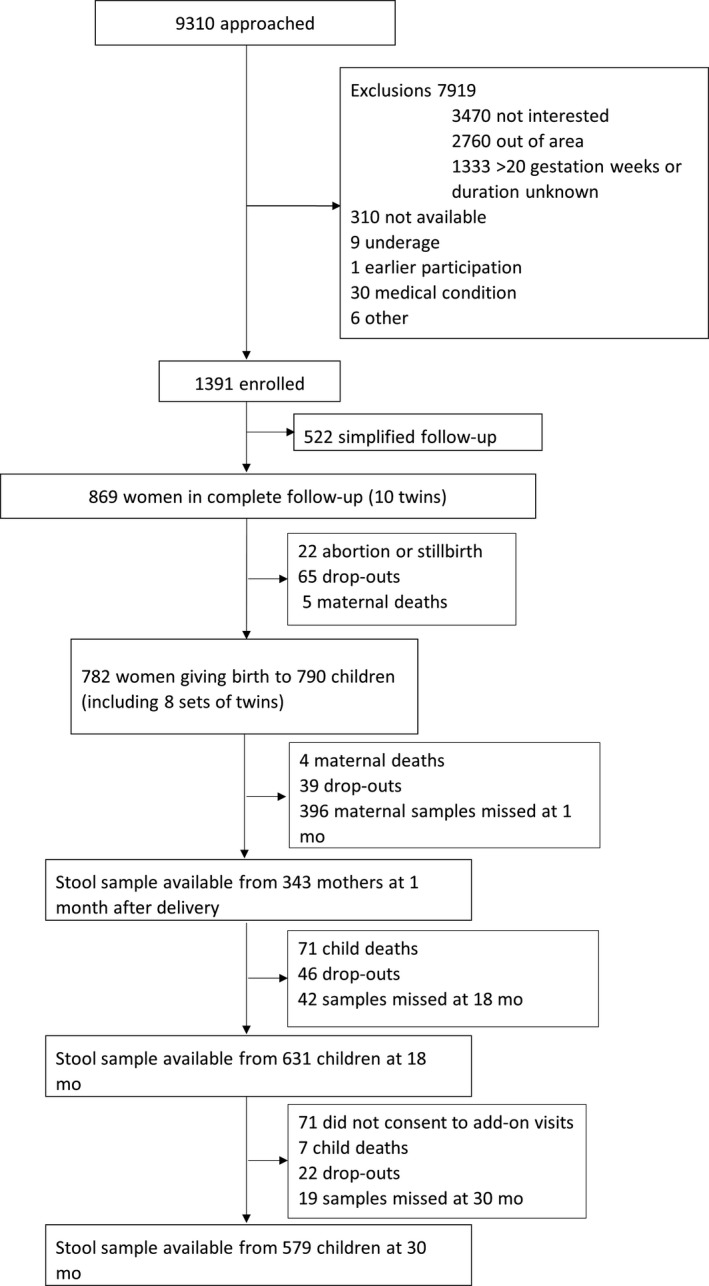
Participant flow

There were 790 liveborn infants including 8 pairs of twins in the complete follow‐up cohort of the iLiNS‐DYAD study. By 30 months, a total of 68 children were lost to follow‐up, 78 had died, and 71 did not consent to extended follow‐up beyond 18 months. Microbiota data were available for 631 participants at 18 months and for 579 participants at 30 months. Details on participant flow are shown in Figure 1.

On average, children included in the analyses were born at 39.5‐week gestation with a birth length of 49.7 cm and their mothers were 25 years of age at enrolment. Most did not have access to piped water or improved sanitary facilities. Most of the baseline characteristics were similar in participants included in and excluded from the analyses. Compared with participants who did not provide microbiota data, the included child participants were born at a higher gestational age, had higher length‐for‐age Z‐score at birth, and their households had a lower assets index and were less likely to have access to piped water (Table 1). The exposure variables duration of exclusive breast feeding and household crowding had more than 20% missing observations and were excluded from primary analyses. Missing data on all other exposure variables combined were <10% of observations at all time points (Table S2).

**Table 1:** Characteristics of included and excluded study participants

Characteristic	Included	Excluded
Participants, n	673	206
Maternal age at enrolment, years	25 (20;29)	23 (19;28)
Maternal education completed, years	3 (0;6)	4 (2;8)
Positive malaria RDT of the mother at enrolment	23%	23%
Gestational age at birth, weeks	39.6 (38.1;40.6)	39.4 (37.4; 40.6)
LAZ at birth	−1.0 (1.1)	−1.4 (1.2)
Length at birth	49.7 (2.3)	49.2 (2.3)
LAZ at 18 mo	−1.7 (1.1)	−2.2 (1.8)
Length at 18 mo	76.9 (3.0)	75.5 (5.8)
Household assets Z‐score	−0.1 (1.0)	0.4 (1.2)
Ownership of any chicken	55%	46%
Ownership of any goats	29%	24%
Ownership of any cows	5%	1%
Source of drinking water is borehole, well, river, or lake (vs piped)	86%	68%
Type of sanitary facility is none or regular pit latrine (vs ventilation improved pit latrine or water closet)	90%	89%

Values are in mean or median (standard deviation or interquartile range) or percentages. *P*‐values are obtained from Mann‐Whitney test or *t* test (continuous variables) or chi‐square test (proportions).

Abbreviations: LAZ, length‐for‐age Z‐score; RDT, rapid diagnostic test.

**Figure 2:**
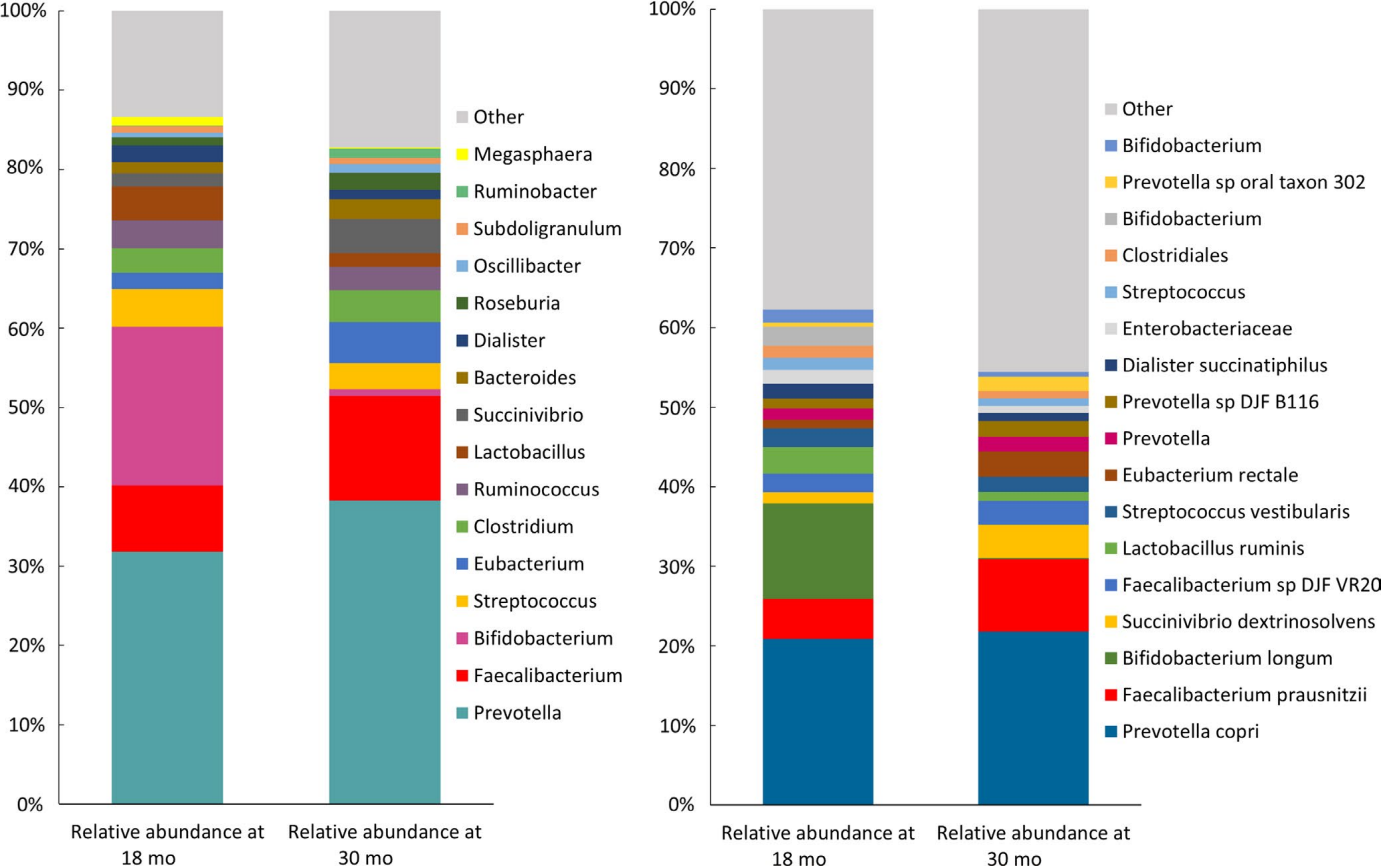
Genera and OTUs with highest relative abundances as percentage of all reads at 18 and 30 mo [Colour figure can be viewed at http://wileyonlinelibrary.com]

The mean (SD) microbiota‐for‐age Z‐score (MAZ) was −1.32 (1.79) at 18 months and −3.7 (2.55) at 30 months. The mean (SD) Shannon index was 2.94 (0.63) at 18 months and 3.53 (0.46) at 30 months. The two outcome measures were strongly correlated with each other at 18 months (*r* = .77) and moderately correlated at 30 months (*r* = .44). Details on microbiota composition at 18 and 30 months are shown in Figure 2. At both time points, the dominant bacterial genus was Prevotella. At 18 months, the second and third most common genera were Bifidobacterium and Faecalibacterium, whereas at 30 months they were Faecalibacterium and Eubacterium.

### 3.2 Microbiota maturity and diversity

There were no significant interactions between predictor variables and intervention group in models predicting MAZ scores or Shannon index, so participants from the three intervention groups were subsequently analysed together. There was no confounding by gestational age at any time point, though gestational age (in weeks) was independently associated with lower microbiota diversity in children at 1 month (ß = −0.06, 95% CI −0.1, −0.01) and higher microbiota maturity and diversity at 6 months (ß = 0.12, 95% CI 0.02, 0.22) for maturity and ß = 0.03, 95% CI 0.002, 0.06) for diversity). Children with maternal education below median had on average higher microbiota maturity and diversity at 18 and 30 months. This association was also observed at 6 months (ß = 0.46, 95% CI 0.11, 0.82) for maturity and ß = 0.14, 95% CI 0.04, 0.24) for diversity), but not at 12 months. Ownership of goats was inversely associated with Shannon index at 18 months and being born by caesarean section was positively associated with Shannon index at 30 months. Results on associations between other environmental exposures and MAZ score or Shannon index at 18 or 30 months are listed in Table 2 and Table S3. Not having access to piped water was positively associated with Shannon index in mothers with similar results in children at 30 months (Table S4). A sensitivity analysis with multiple imputation of missing data for all exposure variables yielded similar results to the analyses without imputed data (Tables S5‐S7).

**Table 2:** The association between environmental exposure variables and the study participants’ microbiota diversity and maturity at 18 mo

		Association between exposure and outcome variable*	
Exposure variables		MAZ score	Shannon index
		Regression coefficient (95% confidence interval)	Regression coefficient (95% confidence interval)
Household assets Z‐score below median		−0.1 (−0.5, 0.3)	0.0 (−0.1, 0.1)
Ownership of any chicken		−0.1 (−0.4, 0.2)	0.0 (−0.1, 0.1)
Ownership of any goats		−0.2 (−0.5, 0.2)	−0.2 (−0.3, −0.0)
Ownership of any cows		−0.1 (−0.8, 0.6)	−0.0 (−0.2, 0.2)
Source of drinking water is borehole, well, river, or lake (vs piped)		−0.0 (−0.5, 0.5)	−0.1 (−0.3, 0.1)
Type of sanitary facility is none or regular pit latrine (vs ventilation improved pit latrine or water closet)		−0.1 (−0.6, 0.5)	−0.0 (−0.2, 0.2)
Antibiotics use above median		−0.2 (−0.5, 0.2)	−0.1 (−0.2, 0.0)
Education level of the mother below median		0.4 (0.0, 0.7)	0.2 (0.1, 0.3)
Age of the mother in years		0.0 (−0.0, 0.0)	0.0 (−0.0, 0.0)
Marital status of the mother is single, divorced, or widowed		−0.2 (−0.7, 0.3)	0.1 (−0.1, 0.3)
Mother HIV positive		0.3 (−0.2, 0.8)	0.0 (−0.1, 0.2)
Male sex of child		0.2 (−0.1, 0.5)	0.0 (−0.1, 0.1)
Delivered by caesarean section		−0.2 (−0.8, 0.5)	0.0 (−0.2, 0.2)
Season	Rainy	0.0 (Reference)	0.0 (Reference)
	Cold dry	0.2 (−0.2, 0.7)	0.1 (−0.1, 0.2)
	Hot dry	−0.3 (−0.7, 0.2)	−0.1 (−0.2, 0.1)

Note

Results from multivariable analysis.

Abbreviation: MAZ score, microbiota‐for‐age Z‐score.

* Adjusted for listed exposure variables, exact age, sample processing pool, and sequencing depth.

### 3.3 Microbial community composition

PERMANOVA revealed that, in 18 months old children, unweighted UniFrac distances were associated with maternal age (*P* = .027), maternal education (*P* = .003), and season (*P* = .005), and weighted UniFrac distances were associated with season (*P* = .046) and ownership of goats (*P* = .043). At 30 months, unweighted UniFrac distances were associated with maternal education (*P* = .006), ownership of goats (*P* = .041), and season (*P* = .011). Further, season was associated with unweighted UniFrac distance at 1 (*P* = .011), 6 (*P* = .013), and 12 months (*P* = .049). In mothers, both unweighted and weighted UniFrac distances were associated with HIV (*P* = .001 and *P* = .013) and water source (*P* = .011 and *P* = .04), and unweighted UniFrac distances were associated with education (*P* = .002) and weighted UniFrac distances with household assets (*P* = .022).

### 3.4 Relative abundances of individual bacterial taxa and genera

**Figure 3:**
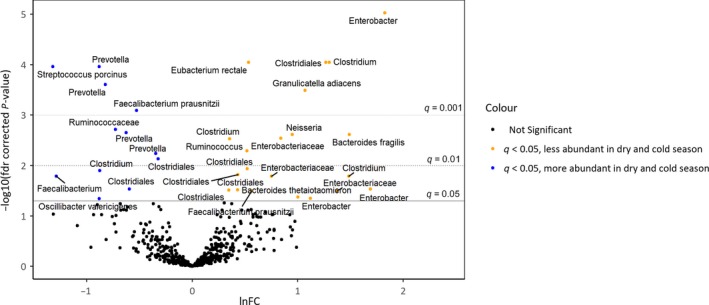
Differences in OTU abundances at 30 mo between samples collected in the dry and cold season and samples collected in the dry and hot or rainy seasons. Results from zero‐inflated negative binominal model adjusted for other environmental predictor variables and covariates. The dots represent OTUs arranged according to ln fold change (lnFC) in OTU abundance between samples collected in different seasons and the corresponding fdr‐corrected *P*‐values. Positive lnFC values represent OTUs that are less abundant in samples collected in the dry and cold season compared with the dry and hot and rainy seasons. Differences in OTUs in orange and blue above the solid line were significant at level q = 0.05 [Colour figure can be viewed at http://wileyonlinelibrary.com]

In analyses of differences in microbiota composition at the taxon level, several OTUs were associated with environmental exposures and other predictor variables. In total, season of stool sample collection (dry and cold season compared with dry and hot and rainy seasons) was associated with most differences in OTU abundances (Figure 3 and Figure S3). Besides season, most associations were observed for ownership of chickens, type of water source, and maternal education. The results for all OTUs included in the analyses grouped by exposure variable are listed in a supplemental table (Table S8). In mothers, 103 OTUs were associated with HIV and among other exposures, and most associations were observed for water source, marital status, and delivery mode.

At the genus level, not having piped water was inversely associated with relative abundances of Roseburia (β = −0.53 SD, 95% CI −0.86, −0.21) and Veillonella (β = −0.37 SD, 95% CI −0.6, −0.12), marital status of the mother (single, divorced, or widowed) was associated with relative abundance of Parabacteroides (β = 0.27 SD, 95% CI 0.14, 0.4), low maternal education was associated with relative abundance of Campylobacter (β = 0.28 SD, 95% CI 0.11, 0.44), and the cold and dry season was associated with lower relative abundances of Megasphaera (ß = −0.36 SD, 95% CI −0.62, −0.09) and higher relative abundance of Ruminococcus (ß = 0.31 SD, 95% CI 0.09, 0.53) in 18‐month‐old children. Not having piped water was also associated with relative abundances of Oscillibacter (ß = 0.62 SD, 95% CI 0.26, 0.98) and Treponema (ß = 0.36 SD, 95% CI 0.13, 0.6), low education was inversely associated with Parabacteroides (ß = −0.38 SD, 95% CI −0.61, −0.16), and HIV was associated with Eubacterium (ß = 0.43 SD, 95% CI 0.13, 0.72), Ruminococcus (ß = 0.55 SD, 95% CI 0.14, 0.96), and taxa of the family Erysipelotrichaceae (ß = 0.48 SD, 95% CI 0.16, 0.8) in mothers.

## 4 COMMENT

### 4.1 Principal findings

This study analysed associations between several environmental exposures and gut microbiota composition in young children and their mothers in rural Malawi. Our findings support an association between gut microbiota composition and season and socio‐economic status in rural Malawi, but do not support the hypothesis that adverse environmental exposures are linked to decreased gut microbiota maturity and diversity. The largest number of associations was in children observed for season, maternal education, and water source, whereas in mothers, most differences were related to HIV status. In general, the average gut microbiota composition in children in our study sample was comparable to that in previous studies in similar populations that have reported microbiota compositions dominated by the genus Prevotella.^11^

### 4.2 Strengths of the study

Strengths of this study were the relatively large sample size, an intensive follow‐up, and standardized and rapid stool sample collection and processing.

### 4.3 Limitations of the data

Our study was limited by missing data on dietary patterns, duration of breast feeding, and household crowding, which are known to influence microbiota composition.^9^, ^13^, ^26^ Sensitivity analyses after multiple imputation of missing data on household crowding and breast feeding gave almost identical results to analyses without these variables, and previous studies in the study area have shown that breast feeding is almost universal through 18 months and that complementary foods mostly consist of a maize‐based porridge with small amounts of other foods.^27^, ^28^ Therefore, we believe that the results are not biased by differences in household crowding or nutrition. As a further limitation, about 10% of the participants were lost to follow‐up, mostly because they moved or were unwilling to continue in the study. In general, participants who did not provide data for this study had higher socio‐economic status than participants from whom data were analysed, but the differences were small and are unlikely to affect the validity of our results. This study only included characteristics of the living environment and basic maternal factors as exposure variables, but it is possible that unmeasured maternal factors like stress and exposure to violence could have an influence on children's gut microbiota in this population.^29^, ^30^, ^31^

### 4.4 Interpretation

To our knowledge, few studies have previously examined seasonal variations in human gut microbiota. Those studies reported seasonal variations likely associated with changes in diet.^18^, ^32^, ^33^ In our study, the observed seasonal differences may be due to shifts in diet, as the dry and cool season follows the harvesting period, but changes in water quality over the year might also be relevant. Socio‐economic factors and biodiversity of the living environment have previously been associated with microbiota.^12^, ^34^ These reports are consistent with our study in which maternal education and type of water source, which can be seen as proxies for socio‐economic status, were associated with different aspects of microbiota composition in both children and their mothers. The impact of water source has not been studied previously; to understand whether there could be a direct link with the microbiota, the differences in quality of water from different sources such as pipe, borehole, or well would need to be examined.

Associations between ownership of domestic animals and microbiota composition have so far mostly been studied for furred pets, and the results have been inconclusive.^13^, ^14^, ^35^, ^36^ We found several associations between ownership of goats and microbiota composition, but only a few associations with ownership of chickens or cows. Because domestic animals, especially chickens and goats, are often housed within household compounds and in close proximity to cooking facilities, it is conceivable that they directly affect the microbial environment that children are exposed to.^37^, ^38^

Delivery mode and antibiotics use have been emphasized as two of the most important factors for gut microbiota development.^9^, ^24^, ^39^, ^40^, ^41^, ^42^, ^43^, ^44^, ^45^, ^46^ We did not find consistent evidence for associations between microbiota and delivery mode, but participants with higher antibiotic intake had on average lower microbiota maturity and diversity. It is not clear how long differences in microbiota caused by delivery mode are sustained, and hence, any earlier differences might have not been detectable at the time points studied.^36^, ^47^ Consistent with earlier reports, HIV status was an important predictor of microbiota composition in mothers in our study sample, though there was no clear association with microbiota diversity.^48^, ^49^, ^50^ We did not conduct HIV tests for children and maternal HIV status showed only few associations with the children's microbiota.

Notably, our study did not find consistent differences in microbiota maturity and diversity related to the direct living environment, but we observed an inverse association with maternal education. There was considerable variation within our study sample in the outcome measures for microbiota maturity and diversity, but it is possible that these measures are too broad to capture specific differences in microbiota composition that are related to environmental factors. Several taxa that were previously found to be age discriminatory in this population were associated with the predictor variables studied, which may indicate that changes over time in microbiota composition are influenced by environmental factors.^8^ Improved understanding about the underlying mechanisms could help to improve health outcomes, also considering that improved living conditions and urbanization can lead to shifts in microbiota composition that are associated with non‐communicable diseases.^51^, ^52^, ^53^, ^54^, ^55^ This is highlighted by the fact that maternal education showed an inverse association with microbiota diversity, suggesting that improved educational levels may have a negative impact on gut microbiota composition. However, further research is needed to determine what constitutes a healthy microbiota composition in this population and what direct or indirect interventions could be beneficial to the developing gut microbiota. Our findings suggest that public health interventions targeting water quality could have an indirect effect on microbiota as water source was associated with several microbiota outcomes.

### 4.5 Conclusions

These findings, together with those from others, suggest that environmental factors in the environment do have an impact on gut microbiota composition, but that there is great contextual variation in this association. In this study in a rural Malawian setting, environmental exposures were associated with several subtle aspects of microbiota composition, but we did not find consistent associations with microbiota maturity or diversity.

## CONFLICT OF INTEREST

The authors declare no conflict of interest.
