# Supplementary figures and images for: Environmental exposures and child and maternal gut microbiota in rural Malawi
Source: Paediatr Perinat Epidemiol. 2020 Feb 3;34(2):161–70. doi: 10.1111/ppe.12623 (PMC7154550; doi:10.1111/ppe.12623)

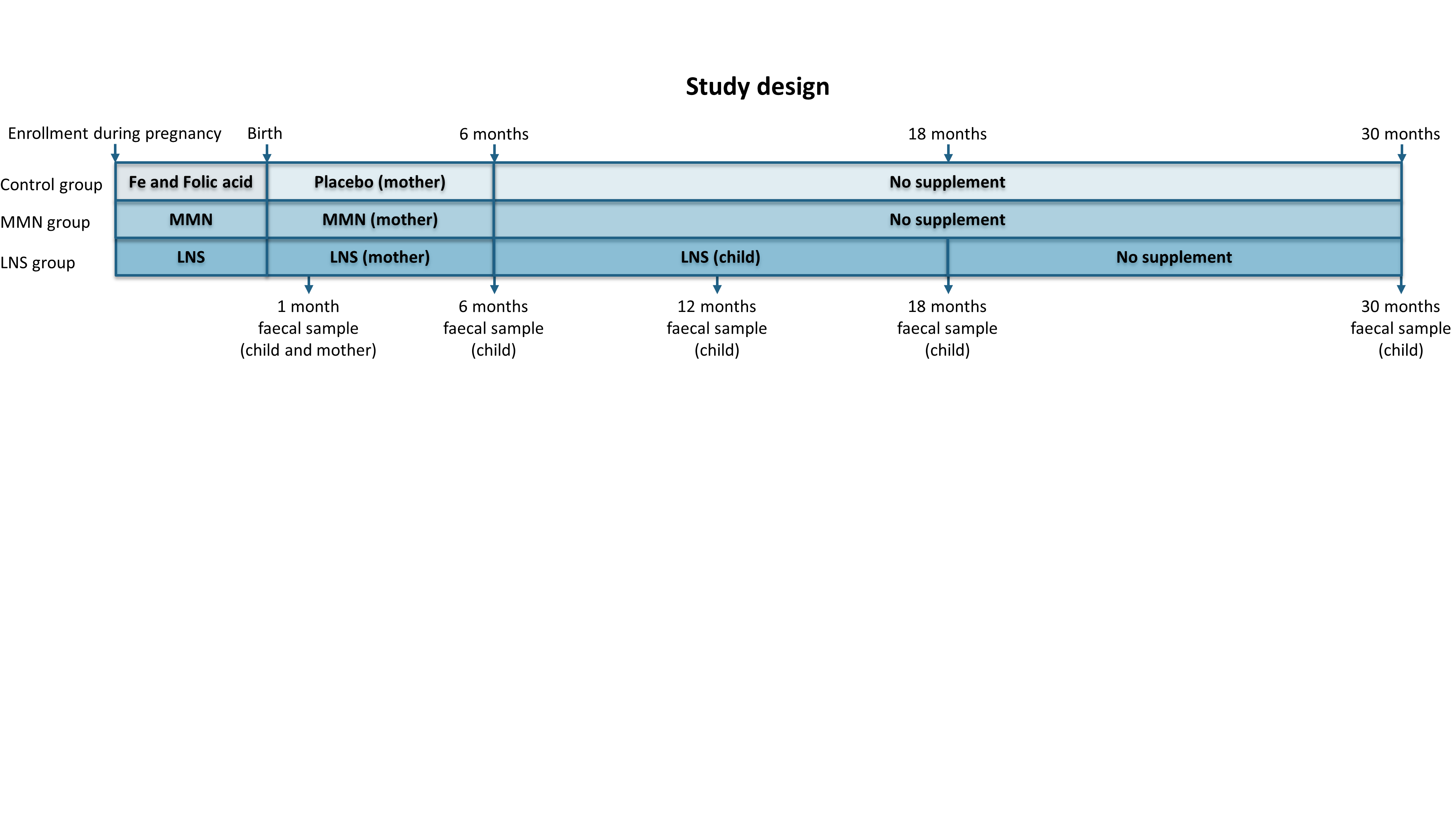

Supplement: Supplementary file 1 [file PPE-34-161-s001.tif]

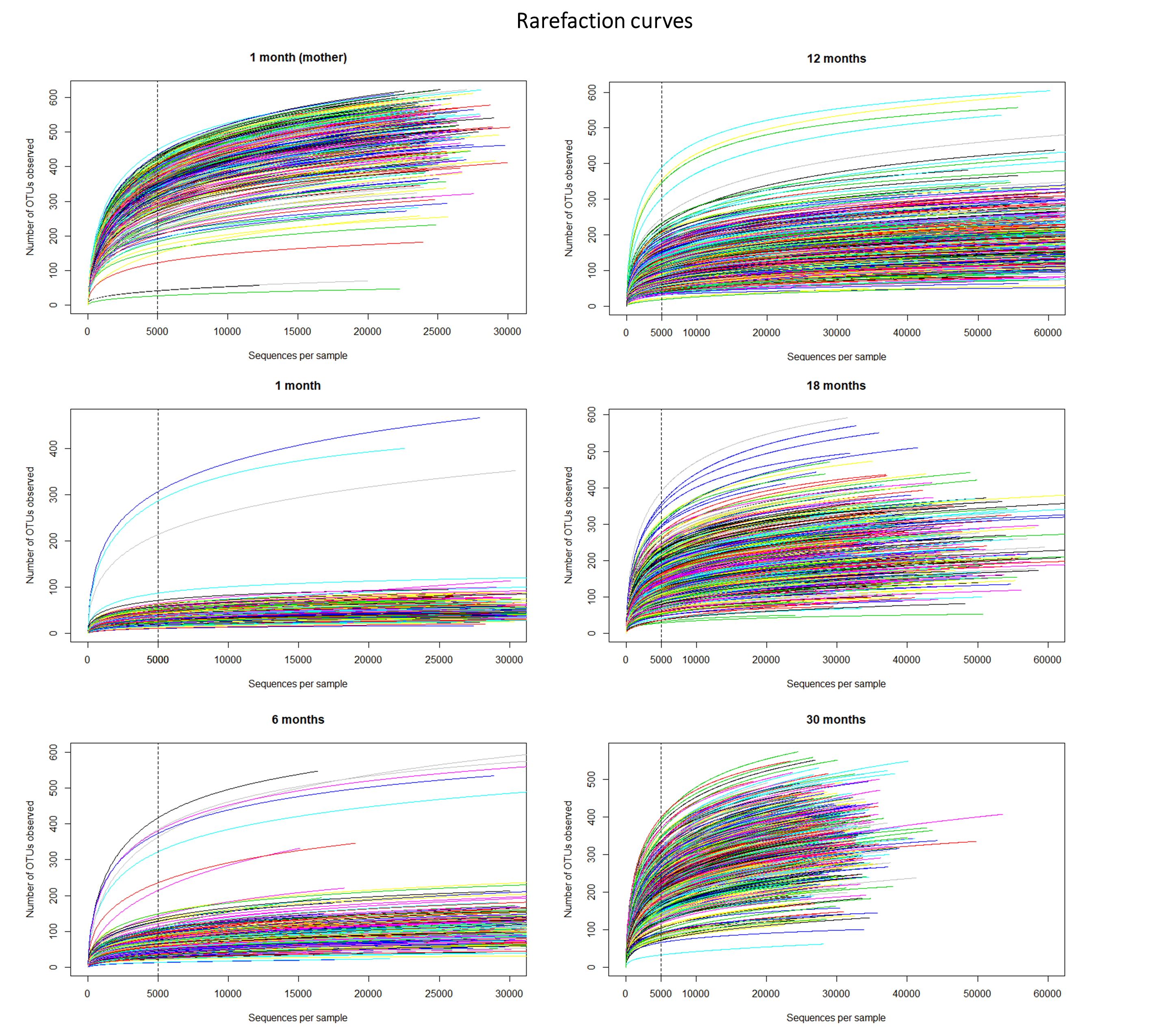

Supplement: Supplementary file 2 [file PPE-34-161-s002.tif]

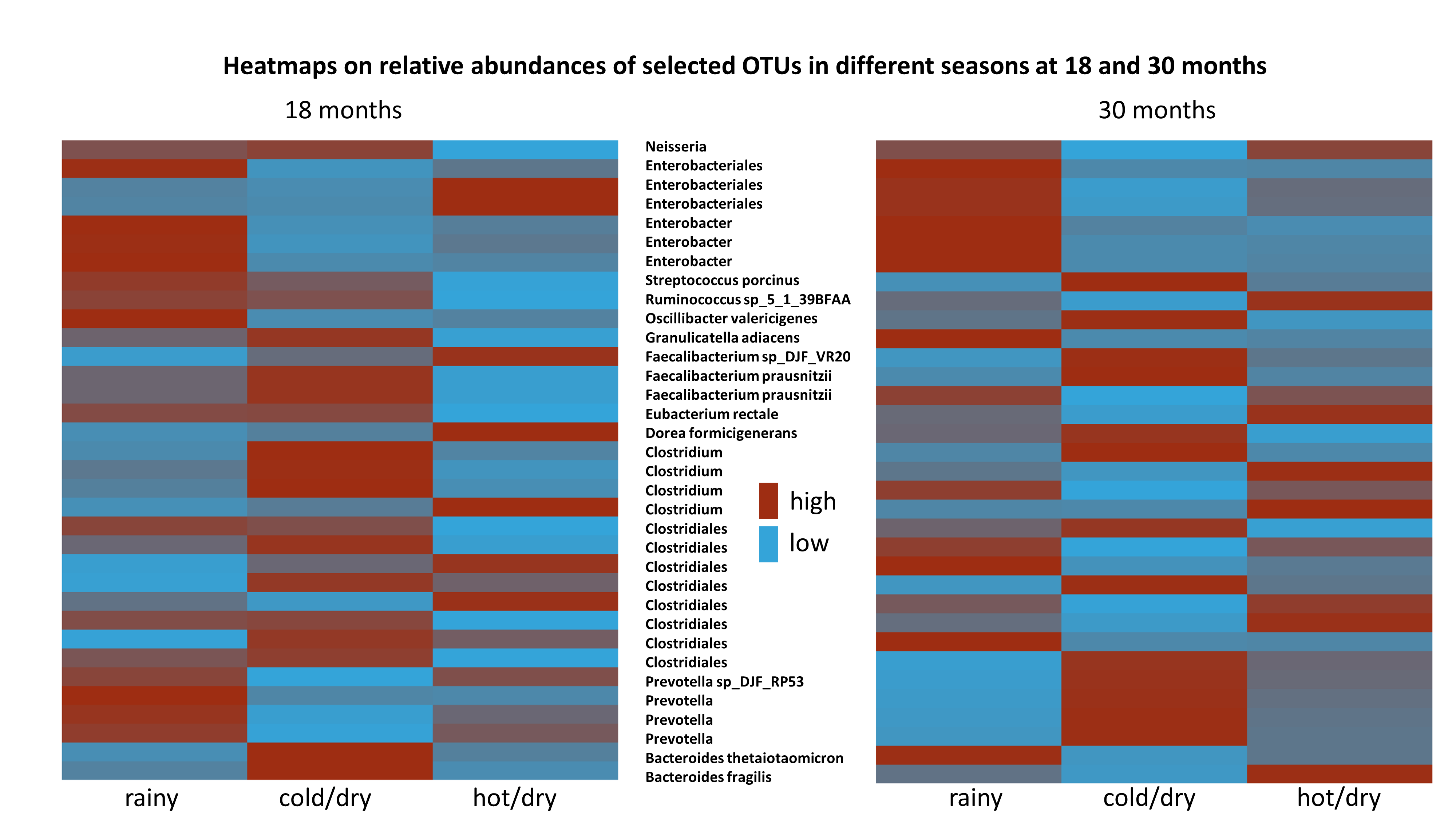

Supplement: Supplementary file 3 [file PPE-34-161-s003.tif]
