# Supplementary material for: Environmental exposures and child and maternal gut microbiota in rural Malawi
Source: Paediatr Perinat Epidemiol. 2020 Feb 3;34(2):161–70. doi: 10.1111/ppe.12623 (PMC7154550; doi:10.1111/ppe.12623)
Supplement: Supplementary file 4 [file PPE-34-161-s004.docx]

**Supplemental tables**

Supplemental Table 1. Inclusion and exclusion criteria for participation in the iLiNS-DYAD Malawi trial

Inclusion criteria:

- Ultrasound confirmed pregnancy of no more than 20 completed gestation weeks
- Permanent resident of Mangochi District Hospital, Malindi Hospital, or Lungwena Health Centre catchment area
- Availability during the period of the study
- Signed informed consent

Exclusion criteria:

- Less than 15 years of age
- Need for frequent medical attention due to a chronic health condition
- Diagnosed asthma treated with regular medication
- Severe illness warranting hospital referral
- History of allergy towards peanuts
- History of anaphylaxis or serious allergic reaction to any substance, requiring emergency medical care
- Pregnancy complications evident at enrolment visit (moderate to severe oedema, blood Hb concentration < 5 g / dl, systolic blood pressure (BP) > 160 mmHg or diastolic BP > 100 mmHg)
- Earlier participation in the iLiNS-DYAD-M trial
- Concurrent participation in any other clinical trial

Supplemental Table 2. Number of missing observations that were imputed for individual exposure variables

|  | **Number of observations for which the exposure variable was imputed at each time point** | | |
| --- | --- | --- | --- |
| **Time point** | 18 months | 30 months | 1 month maternal data |
| n total | 622 | 574 | 343 |
| Household assets Z-score below median | 0 | 0 | 0 |
| Ownership of any chicken | 24 | 26 | 9 |
| Ownership of any goats | 24 | 26 | 9 |
| Ownership of any cows | 24 | 26 | 9 |
| Source of drinking water is borehole, well, river or lake (vs. piped) | 0 | 1 | 23 |
| Type of sanitary facility is none or regular pit latrine (vs. ventilation improved pit latrine or water closet) | 0 | 1 | 23 |
| Antibiotics use above median | 3 | 4 | - |
| Education level of the mother below median | 3 | 4 | 3 |
| Age of the mother in years | 0 | 0 | 0 |
| Marital status of the mother is single, divorced or widowed | 1 | 2 | 0 |
| Mother HIV positive | 3 | 1 | 1 |
| Male sex of child | 0 | 0 | - |
| Delivered by caesarean section | 8 | 6 | 28 |
| Duration of exclusive breast feeding (in weeks) | 277 | 249 | - |
| Household crowding (number of people living in the household) | 141 | 125 | 113 |

Supplemental Table 3. The association between environmental exposure variables and the study participants’ microbiota diversity and maturity at 30 months. Results from multivariable analysis.

|  | | Association between exposure and outcome variables * | |
| --- | --- | --- | --- |
| Exposure variables | | MAZ-score^a^ | Shannon Index |
|  |  | Regression coefficient (95% confidence interval) | Regression coefficient (95% confidence interval) |
| Household assets Z-score below median | | -0.0 (-0.6, 0.5) | -0.0 (-0.1, 0.1) |
| Ownership of any chicken | | 0.2 (-0.3, 0.6) | 0.0 (-0.1, 0.1) |
| Ownership of any goats | | 0.1 (-0.4, 0.6) | 0.0 (-0.1, 0.1) |
| Ownership of any cows | | -0.9 (-1.9, 0.2) | -0.1 (-0.3, 0.1) |
| Source of drinking water is borehole, well, river or lake (vs. piped) | | 0.5 (-0.3, 1.3) | 0.1 (-0.0, 0.3) |
| Type of sanitary facility is none or regular pit latrine (vs. ventilation improved pit latrine or water closet) | | 0.2 (-0.7, 1.0) | 0.0 (-0.1, 0.2) |
| Antibiotics use above median | | -0.4 (-0.8, 0.1) | -0.0 (-0.1, 0.1) |
| Education level of the mother below median | | 0.5 (0.0, 0.9) | 0.1 (0.0, 0.2) |
| Age of the mother in years | | -0.0 (-0.0, 0.0) | -0.0 (-0.0, 0.0) |
| Marital status of the mother is single, divorced or widowed | | 0.0 (-0.7, 0.7) | -0.0 (-0.2, 0.1) |
| Mother HIV positive | | 0.0 (-0.7, 0.7) | 0.0 (-0.1, 0.2) |
| Male sex of child | | -0.2 (-0.7, 0.2) | -0.0 (-0.1, 0.1) |
| Delivered by caesarean section | | 0.3 (-0.7, 1.2) | 0.2 (0.0, 0.3) |
| Season | rainy | 0.0 (Reference) | 0.0 (Reference) |
|  | cold dry | 0.2 (-0.3, 0.8) | -0.1 (-0.2, 0.0) |
|  | hot dry | 0.3 (-0.3, 0.9) | -0.0 (-0.1, 0.1) |

a) MAZ-score, microbiota-for-age Z-score

*adjusted for listed exposure variables, exact age, and sequencing depth

Supplemental Table 4. The association between environmental exposure variables and maternal microbiota diversity at 1 month after delivery. Results from multivariable analysis.

|  | | **Association between exposure and outcome variable *** |
| --- | --- | --- |
| **Exposure variables** | | Shannon Index |
|  |  | Regression coefficient (95% confidence interval) |
| Household assets Z-score below median | | -0.1 (-0.2, 0.1) |
| Ownership of any chicken | | -0.2 (-0.2, 0.1) |
| Ownership of any goats | | 0.0 (-0.1, 0.1) |
| Ownership of any cows | | 0.1 (-0.2, 0.4) |
| Source of drinking water is borehole, well, river or lake (vs. piped) | | 0.4 (0.1, 0.6) |
| Type of sanitary facility is none or regular pit latrine (vs. ventilation improved pit latrine or water closet) | | -0.1 (-0.3, 0.2) |
| Education level below median | | 0.0 (-0.1, 0.2) |
| Age in years | | 0.0 (-0.0, 0.0) |
| Marital status is single, divorced or widowed | | 0.1 (-0.1, 0.3) |
| HIV positive | | -0.1 (-0.3, 0.1) |
| Delivered by caesarean section | | -0.3 (-0.6, -0.0) |
| Season | rainy | 0.0 (Reference) |
|  | cold dry | -0.1 (-0.2, 0.1) |
|  | hot dry | -0.0 (-0.2, 0.2) |

a) MAZ-score, microbiota-for-age Z-score

* adjusted for listed exposure variables and sequencing depth

Supplemental Table 5. The association between environmental exposure variables and the study participants’ microbiota diversity and maturity at 18 months. Results from multivariable analysis with multiple imputation of missing exposure variables.

|  | | **Association between exposure and outcome variable *** | | | |
| --- | --- | --- | --- | --- | --- |
| **Exposure variables** | | | MAZ-score^a^ | Shannon Index | |
|  |  |  | Regression coefficient (95% confidence interval) | Regression coefficient (95% confidence interval) | |
| Household assets Z-score below median | | | -0.1 (-0.5, 0.3) | -0.0 (-0.2, 0.1) | |
| Ownership of any chicken | | | -0.1 (-0.4, 0.2) | 0.0 (-0.1, 0.1) | |
| Ownership of any goats | | | -0.1 (-0.5, 0.2) | -0.1 (-0.3, -0.0) | |
| Ownership of any cows | | | -0.0 (-0.7, 0.7) | -0.0 (-0.3, 0.2) | |
| Source of drinking water is borehole, well, river or lake (vs. piped) | | | -0.1 (-0.6, 0.5) | -0.1 (-0.3, 0.1) | |
| Type of sanitary facility is none or regular pit latrine (vs. ventilation improved pit latrine or water closet) | | | -0.1 (-0.6, 0.5) | -0.0 (-0.2, 0.2) | |
| Antibiotics use above median | | | -0.2 (-0.5, 0.2) | -0.1 (-0.2, 0.1) | |
| Education level of the mother below median | | | 0.3 (0.0, 0.7) | 0.2 (0.1, 0.3) | |
| Age of the mother in years | | | 0.0 (-0.0, 0.0) | 0.0 (-0.0, 0.0) | |
| Marital status of the mother is single, divorced or widowed | | | -0.3 (-0.7, 0.2) | 0.1 (-0.1, 0.2) | |
| Mother HIV positive | | | 0.3 (-0.1, 0.8) | 0.0 (-0.2, 0.2) | |
| Male sex of child | | | 0.2 (-0.1, 0.5) | 0.0 (-0.1, 0.1) | |
| Delivered by caesarean section | | | -0.1 (-0.7, 0.5) | 0.0 (-0.2, 0.2) | |
| Season | rainy | | 0.0 (Reference) | 0.0 (Reference) | |
|  | cold dry | | 0.3 (-0.2, 0.7) | 0.1 (-0.1, 0.2) | |
|  | hot dry | | -0.3 (-0.7, 0.1) | -0.1 (-0.2, 0.1) | |
| Duration of exclusive breast feeding (in weeks) | | | 0.0 (-0.0, 0.0) | 0.0 (-0.0, 0.0) | |
| Household crowding (number of people living in the household) | | | 0.0 (-0.0, 0.1) | | 0.0 (-0.0, 0.0) |

a) MAZ-score, microbiota-for-age Z-score

* adjusted for listed exposure variables, exact age, sample processing pool, and sequencing depth

Supplemental Table 6. The association between environmental exposure variables and the study participants’ microbiota diversity and maturity at 30 months. Results from multivariable analysis with multiple imputation of missing exposure variables.

|  | | **Association between exposure and outcome variable *** | | | |
| --- | --- | --- | --- | --- | --- |
| **Exposure variables** | | | MAZ-score^a^ | Shannon Index | |
|  |  |  | Regression coefficient (95% confidence interval) | Regression coefficient (95% confidence interval) | |
| Household assets Z-score below median | | | 0.0 (-0.5, 0.6) | 0.0 (-0.1, 0.1) | |
| Ownership of any chicken | | | 0.1 (-0.3, 0.6) | 0.0 (-0.1, 0.1) | |
| Ownership of any goats | | | 0.1 (-0.4, 0.6) | 0.0 (-0.1, 0.1) | |
| Ownership of any cows | | | -0.9 (-2.0, 0.1) | -0.1 (-0.3, 0.1) | |
| Source of drinking water is borehole, well, river or lake (vs. piped) | | | 0.5 (-0.3, 1.3) | 0.1 (-0.0, 0.3) | |
| Type of sanitary facility is none or regular pit latrine (vs. ventilation improved pit latrine or water closet) | | | 0.2 (-0.6, 1.0) | 0.0 (-0.1, 0.2) | |
| Antibiotics use above median | | | -0.4 (-0.8, 0.0) | -0.0 (-0.1, 0.1) | |
| Education level of the mother below median | | | 0.5 (0.0, 0.9) | 0.1 (0.0, 0.2) | |
| Age of the mother in years | | | -0.0 (-0.0, 0.0) | 0.0 (-0.0, 0.0) | |
| Marital status of the mother is single, divorced or widowed | | | -0.0 (-0.7, 0.0) | -0.0 (-0.2, 0.1) | |
| Mother HIV positive | | | -0.1 (-0.7, 0.6) | 0.0 (-0.1, 0.2) | |
| Male sex of child | | | -0.3 (-0.7, 0.2) | 0.0 (-0.1, 0.1) | |
| Delivered by caesarean section | | | 0.3 (-0.6, 1.0-3) | 0.2 (-0.0, 0.3) | |
| Season | rainy | | 0.0 (Reference) | 0.0 (Reference) | |
|  | cold dry | | 0.3 (-0.3, 0.8) | -0.1 (-0.2, 0.0) | |
|  | hot dry | | 0.3 (-0.4, 0.9) | -0.0 (-0.1, 0.1) | |
| Duration of exclusive breast feeding (in weeks) | | | 0.0 (-0.0, 0.1) | 0.0 (-0.0, 0.0) | |
| Household crowding (number of people living in the household) | | | -0.0 (-0.1, 0.1) | | -0.0 (-0.0, 0.0) |

a) MAZ-score, microbiota-for-age Z-score

* adjusted for listed exposure variables, exact age, and sequencing depth

Supplemental Table 7. The association between environmental exposure variables and maternal microbiota diversity at 1 month after delivery. Results from multivariable analysis with multiple imputation of missing exposure variables.

|  | | **Association between exposure and outcome variable *** |
| --- | --- | --- |
| **Exposure variables** | | Shannon Index |
|  |  | Regression coefficient (95% confidence interval) |
| Household assets Z-score below median | | -0.1 (-0.4, 0.1) |
| Ownership of any chicken | | 0.0 (-0.1, 0.1) |
| Ownership of any goats | | 0.0 (-0.1, 0.2) |
| Ownership of any cows | | 0.1 (-0.2, 0.4) |
| Source of drinking water is borehole, well, river or lake (vs. piped) | | 0.3 (0.0, 0.5) |
| Type of sanitary facility is none or regular pit latrine (vs. ventilation improved pit latrine or water closet) | | -0.0 (-0.3, 0.2) |
| Education level below median | | -0.1 (-0.2, 0.1) |
| Age in years | | 0.0 (-0.0, 0.0) |
| Marital status is single, divorced or widowed | | 0.1 (-0.1, 0.3) |
| HIV positive | | -0.1 (-0.3, 0.1) |
| Delivered by caesarean section | | -0.2 (-0.4, 0.1) |
| Season | rainy | 0.0 (Reference) |
|  | cold dry | -0.1 (-0.2, 0.1) |
|  | hot dry | -0.0 (-0.2, 0.0) |
| Household crowding (number of people living in the household) | | -0.0 (-0.0, 0.0) |

a) MAZ-score, microbiota-for-age Z-score

* adjusted for listed exposure variables and sequencing depth
