# Supplementary material for: Environmental exposures and child and maternal gut microbiota in rural Malawi
Source: Paediatr Perinat Epidemiol. 2020 Feb 3;34(2):161–70. doi: 10.1111/ppe.12623 (PMC7154550; doi:10.1111/ppe.12623)
Supplement: Supplementary file 6 [file PPE-34-161-s006.docx]

**Supplemental Methods**

Cohort selection

The iLiNS-DYAD trial during which the data for this study were collected was a randomized, controlled, and partly blinded clinical trial that was conducted in the Mangochi district in southern Malawi. The study area is mostly rural with one semi-urban area and has a high prevalence of child undernutrition and high fertility rates. The study enrolled 1391 pregnant women with ultrasound confirmed pregnancy of no more than 20 completed gestation weeks who were permanent residents of the study catchment area. Exclusion criteria included age less than 15 years, severe illness, allergy towards peanuts, pregnancy complications, and participation in any other clinical trial (eTable 1). The first 869 women that were enrolled participated in a complete follow-up scheme and were randomly assigned to receive either LNS or multiple-micronutrient tablets during pregnancy and 6 months postpartum or only iron and folic acid during pregnancy and placebo during 6 months after giving birth.

The children of the mothers who were in the LNS group received LNS between 6 and 18 months of age (eFigure 1). Of these children, 631 were followed for an additional 12 months after the intervention period. The remaining 522 mothers and their children were assigned to a simplified follow-up scheme in which there was no postnatal intervention and data were collected on a limited number of variables. Only data from children and mothers in the complete follow-up scheme were used for this study.

Data and faecal sample collection

Mothers were followed up fortnightly during pregnancy whereas children were followed weekly until the age of 18 months. The weekly visits were conducted at the participants’ homes to assess morbidity symptoms, development and health seeking behaviour. Clinic visits were conducted at 1 week and 6, 12, 18, 24, and 30 months after birth to take anthropometric measurements and collect clinical and socioeconomic data. Faecal samples from children were collected at home visits at 1, 6, 12, 18, and 30 months of age and from mothers at 1 month after delivery (eFigure 1). If a participant had diarrhoea, no sample was collected and the visit was postponed by two weeks.

Samples that had been placed in collection containers by mothers were picked up by research assistants on the same day and placed in cooler bags, after which time they were transferred to cryovial tubes for storage and frozen at -20°C within approximately 6 hours. Within 48 hours, samples were transported to a central laboratory where they were frozen at -80°C and stored for up to six months until being shipped to the analysis laboratory on dry ice.

Sample processing

Microbiota data were obtained from the above mentioned faecal samples using previously described DNA extraction and high-throughput sequencing methods.(1–3) Briefly, the samples were cryo-pulverized using liquid nitrogen, suspended in a liquid containing phenol:chloroform:isoamyl alcohol, and shaken in a bead beater (BioSpec Products, Bartlesville, OK) with 0.1mm silica/zirconia beads to mechanically disrupt bacterial cells. The samples were then centrifuged twice to separate the aqueous phase containing DNA. DNA was purified by precipitation in a high-salt, low pH, isopropanol-containing liquid and binding to a silica membrane that was washed with elution buffer (QIAquick column, Qiagen, Germantown, MD). The amount of DNA in all samples was normalized following fluorometric quantitation (Qubit, Thermo Fisher Scientific, Waltham, MA) by diluting the samples with water. Subsequently, the 16S V4 region of the bacterial DNA was amplified by PCR using primers with a barcode sequence unique to each sample.(4) After a second normalization step and pooling and purification of all samples, the amplicons were sequenced using an Illumina MiSeq instrument (version 2 chemistry, Illumina, San Diego, CA). Paired-end 250bp reads were trimmed to 200bp, combined, and clustered according to 97% base pair identity using QIIME.(5) These clusters were each defined as an operational taxonomic unit (OTU) and sequences were aligned with PyNAST. The Ribosomal Database Project version 2.4 classifier was trained with a custom dataset of bacterial taxonomy and OTUs were assigned taxonomy mostly to genus or species level resolution.(2,6) The relative abundance of each OTU in each sample was quantified by the number of sequence reads assigned to it. To exclude artefacts, OTUs were filtered with a threshold of 0.1% of sequencing reads in at least two samples. The V4-16S sequence data generated and analysed for this study are available through the European Nucleotide Archive under the study accession number PRJEB29433.

Outcomes

Different measures capturing specific aspects of microbiota composition were used as outcome variables. Variables measuring microbiota maturity and diversity in children at 18 and 30 months were used as primary outcomes. Additionally, we performed secondary analyses with microbiota outcomes at 1, 6, and 12 months in children and 1 month after delivery in mothers to assess whether the effect of environmental exposures on child microbiota composition increases with age as children become more mobile and start consuming complementary foods and whether these effects are also observed in their mothers. Other secondary outcomes included variables measuring phylogenetic distances between samples and the relative abundance of individual bacterial OTUs and genera.

Abundance data were rarefied to 5,000 reads per sample before maturity and diversity variables were calculated. There were 17, 14, and 8 samples with less than 5,000 sequencing reads at 6, 18, and 30 months, respectively, that were excluded after rarefaction. (eFigure 2) To measure microbiota maturity, a previously built random forest machine learning model with microbiota data from a reference group of healthy Malawian children was used(1). The model utilized the relative abundances of 25 OTUs found to be age-discriminatory up to 24 months after birth in these healthy children. Microbiota ages of study participants predicted by this random forest model were compared to the median microbiota age of same-aged children in the reference group to obtain microbiota-for-age Z-scores (MAZ-scores). Details on this method have been described previously.(1,7)

To quantify microbiota diversity, Shannon’s diversity Index as a measure of species richness and evenness was used. Microbiota diversity has been shown to increase with age during the first years of life and can thus be seen as an additional measure of microbiota maturity in this study population.(8,9)

MAZ-scores and Shannon’s diversity index are measures that aggregate the microbiota composition into interpretable outcomes. However, they do not allow for conclusions on differences in microbiota composition at the species and genus level, or differences in overall microbiota composition between groups. Therefore, weighted and unweighted UniFrac distances were used to compare overall microbiota composition between participants with different levels of environmental exposures. (10,11) To enable comparisons of the relative abundances of specific bacterial taxa and genera, unrarefied OTU counts that were normalized using cumulative sum-scaling (CSS) were used as outcomes in descriptive analyses.(12)

Exposures

Predictor variables included measures of environmental exposures and other factors that could influence microbiota composition based on previous studies and biological plausibility. As a proxy for socio-economic status, a previously described household assets index was constructed by principal component analysis based on ownership of a set of assets (radio, television, cell phone, bed, mattress, bed net, and bicycle), lighting source, drinking water source, sanitary facility, and flooring materials.(13) Having a household assets index below the sample median was considered an adverse environmental exposure, as was the presence of any chickens, goats, or cows in the household, not having piped drinking water, having no sanitary facility or a regular pit latrine, maternal education below the sample median (in years of primary and secondary school), maternal HIV and maternal marital status other than married. Because delivery mode, duration of exclusive breast feeding, season, age, and antibiotic use have been shown to be associated with microbiota composition, these variables as well as child sex, maternal age, household crowding (in number of people living in the household), sample processing pool and 16S rRNA sequencing depth were analysed as secondary exposures.(8,14–16) Information on socioeconomic variables was collected by trained data collectors through structured interviews during pregnancy whereas antibiotic use was reported by care-givers at weekly home visits between birth and 18 months of age. Season was classified in three categories as rainy for November to April, cool and dry for May to August, and hot and dry for September to October. All exposure variables were checked for multicollinearity.

Statistical analysis

Separate linear models for each time point were used to test the hypotheses that environmental exposures are associated with decreased microbiota maturity (MAZ) and diversity (Shannon’s diversity index). Analyses were performed with multivariable models that included all environmental exposure variables and secondary exposures. Because the participant’s intervention group in the iLiNS-DYAD trial may modify the association between the environmental exposure variables and the outcomes of interest, an interaction term was added to the multivariable models to test for interaction between the trial intervention and each exposure variable. These tests were performed for each outcome at 18 months before proceeding to the main analysis. No interaction tests were performed for outcomes at 30 months because the study intervention finished at 18 months. Additionally, we examined confounding by gestational age at delivery at all time points in the child samples.

Permutational multivariate analysis of variance (PERMANOVA) models using both weighted and unweighted UniFrac distances were used to test whether subjects with similar environmental exposures had microbiota compositions more similar to each other than to those of subjects with different exposures .(17) The models included all environmental exposure variables and covariates as predictors and assessed the marginal effect of each predictor variable. Pseudo p-values were obtained by permutation.

Differences in bacterial relative abundances were analysed at OTU and genus level. Differences in CSS-normalized counts of specific OTUs were tested with multivariable zero-inflated negative binominal models due to the large numbers of zeroes characteristic of datasets of OTU abundances. All predictor variables were included in the analysis. Differences in all OTUs that had non-zero counts in at least 20% of all samples were analysed and FDR-corrected p-values were calculated. Differences in the relative abundances of the 23 most common genera were tested by assessing associations between predictor variables and OTU counts aggregated to genus level using multivariable regression models with robust standard errors.

Analyses were performed in STATA version 13 and in R version 3.2.1 with the package phyloseq.(18)

References:

1. Blanton LV, Charbonneau MR, Salih T et al. Gut bacteria that prevent growth impairments transmitted by microbiota from malnourished children. *Science* 2016;**351**:aad3311–aad3311.

2. Ridaura VK, Faith JJ, Rey FE et al. Gut microbiota from twins discordant for obesity modulate metabolism in mice. *Science* 2013;**341**:1241214.

3. Subramanian S, Huq S, Yatsunenko T et al. Persistent gut microbiota immaturity in malnourished Bangladeshi children. *Nature* 2014;**510**:417–421.

4. Caporaso JG, Lauber CL, Walters WA et al. Global patterns of 16S rRNA diversity at a depth of millions of sequences per sample. *Proc Natl Acad Sci U S A* 2011;**108 Suppl 1**:4516–4522.

5. Caporaso JG, Kuczynski J, Stombaugh J et al. QIIME allows analysis of high-throughput community sequencing data. *Nat Methods* 2010;**7**:335–336.

6. Wang Q, Garrity GM, Tiedje JM et al. Naive Bayesian Classifier for Rapid Assignment of rRNA Sequences into the New Bacterial Taxonomy. *Appl Environ Microbiol* 2007;**73**:5261–5267.

7. Subramanian S, Huq S, Yatsunenko T et al. Persistent gut microbiota immaturity in malnourished Bangladeshi children. *Nature* Published Online First: 4 June 2014. doi:10.1038/nature13421

8. Yatsunenko T, Rey FE, Manary MJ et al. Human gut microbiome viewed across age and geography. *Nature* 2012;**486**:222–227.

9. Avershina E, Storrø O, Øien T et al. Major faecal microbiota shifts in composition and diversity with age in a geographically restricted cohort of mothers and their children. *FEMS Microbiol Ecol* 2014;**87**:280–290.

10. Lozupone C, Knight R. UniFrac: a new phylogenetic method for comparing microbial communities. *Appl Environ Microbiol* 2005;**71**:8228–8235.

11. Lozupone C, Lladser ME, Knights D et al. UniFrac: an effective distance metric for microbial community comparison. *ISME J* 2011;**5**:169–172.

12. Paulson JN, Stine OC, Bravo HC et al. Differential abundance analysis for microbial marker-gene surveys. *Nat Methods* 2013;**10**:1200–1202.

13. Filmer D, Pritchett LH. Estimating wealth effects without expenditure data--or tears: an application to educational enrollments in states of India. *Demography* 2001;**38**:115–132.

14. Levin AM, Sitarik AR, Havstad SL et al. Joint effects of pregnancy, sociocultural, and environmental factors on early life gut microbiome structure and diversity. *Sci Rep* 2016;**6**:31775.

15. Bokulich NA, Chung J, Battaglia T et al. Antibiotics, birth mode, and diet shape microbiome maturation during early life. *Sci Transl Med* 2016;**8**:343ra82-343ra82.

16. Davenport ER, Mizrahi-Man O, Michelini K et al. Seasonal Variation in Human Gut Microbiome Composition. *PLoS ONE* 2014;**9**:e90731.

17. Anderson MJ. A new method for non-parametric multivariate analysis of variance: NON-PARAMETRIC MANOVA FOR ECOLOGY. *Austral Ecol* 2001;**26**:32–46.

18. McMurdie PJ, Holmes S. phyloseq: An R Package for Reproducible Interactive Analysis and Graphics of Microbiome Census Data. *PLoS ONE* 2013;**8**:e61217.
